# Comparison of test–retest reliability of BOLD and pCASL fMRI in a two-center study

**DOI:** 10.1186/s12880-022-00791-9

**Published:** 2022-04-03

**Authors:** James W. Ibinson, Andrea G. Gillman, Vince Schmidthorst, Conrad Li, Vitaly Napadow, Marco L. Loggia, Ajay D. Wasan

**Affiliations:** 1 Department of Anesthesiology and Perioperative Medicine, University of Pittsburgh School of Medicine, University of Pittsburgh, Pittsburgh, PA USA; 2 Department of Radiology, University of Pittsburgh, School of Medicine, University of Pittsburgh, Pittsburgh, PA USA; 3 Department of Bioengineering, Swanson School of Engineering, University of Pittsburgh, Pittsburgh, PA USA; 4 A. A. Martinos Center for Biomedical Imaging, Department of Radiology, Massachusetts General Hospital, Harvard Medical School, Charlestown, MA USA

**Keywords:** Test–retest reliability, Pseudo-continuous arterial spin labeling, Blood oxygen level dependent, Resting state functional connectivity, Finger tapping, Functional magnetic resonance imaging

## Abstract

**Background:**

The establishment of test–retest reliability and reproducibility (TRR) is an important part of validating any research tool, including functional magnetic resonance imaging (fMRI). The primary objective of this study is to investigate the reliability of pseudo-Continuous Arterial Spin Labeling (pCASL) and Blood Oxygen Level Dependent (BOLD) fMRI data acquired across two different scanners in a sample of healthy adults. While single site/single scanner studies have shown acceptable repeatability, TRR of both in a practical multisite study occurring in two facilities spread out across the country with weeks to months between scans is critically needed.

**Methods:**

Ten subjects were imaged with similar 3 T MRI scanners at the University of Pittsburgh and Massachusetts General Hospital. Finger-tapping and Resting-state data were acquired for both techniques. Analysis of the resting state data for functional connectivity was performed with the Functional Connectivity Toolbox, while analysis of the finger tapping data was accomplished with FSL. pCASL Blood flow data was generated using AST Toolbox. Activated areas and networks were identified via pre-defined atlases and dual-regression techniques. Analysis for TRR was conducted by comparing pCASL and BOLD images in terms of Intraclass correlation coefficients, Dice Similarity Coefficients, and repeated measures ANOVA.

**Results:**

Both BOLD and pCASL scans showed strong activation and correlation between the two locations for the finger tapping tasks. Functional connectivity analyses identified elements of the default mode network in all resting scans at both locations. Multivariate repeated measures ANOVA showed significant variability between subjects, but no significant variability for location. Global CBF was very similar between the two scanning locations, and repeated measures ANOVA showed no significant differences between the two scanning locations.

**Conclusions:**

The results of this study show that when similar scanner hardware and software is coupled with identical data analysis protocols, consistent and reproducible functional brain images can be acquired across sites. The variability seen in the activation maps is greater for pCASL versus BOLD images, as expected, however groups maps are remarkably similar despite the low number of subjects. This demonstrates that multi-site fMRI studies of task-based and resting state brain activity is feasible.

## Introduction

Functional brain imaging is a technique commonly used in neuroscience research to measure brain metabolism or blood flow, identifying areas of the brain responsible for functions such as sensation, motor control, and some aspects of cognition [1]. One commonly used technique is blood oxygen level dependent (BOLD) functional magnetic resonance imaging (fMRI), where changes in blood flow (as a surrogate for metabolism changes) can be qualitatively followed due to the differing magnetic susceptibility of oxygenated and deoxygenated hemoglobin [2]. In contrast to the purely qualitative images of the BOLD technique, arterial spin labeling (ASL) is able to provide both qualitative perfusion images similar to BOLD and quantitative measurements of blood flow [3]. The signal from ASL data is primarily localized to the capillary bed, and thus the measured signal should be more closely localized with the neurons of interest [4].

The establishment of test–retest reliability and reproducibility (TRR) is an important part of validating any research tool, fMRI [5, 6]. Investigations of TRR for BOLD-based research have been performed for block design paradigms [7], task-based functional connectivity (fcMRI) [8], and resting state fcMRI (rs-fcMRI) [9]. These have typically shown that the intraclass correlation coefficients (ICCs) are acceptable for both measures of activity and resting state networks (RSNs), with values typically greater than 0.6. TTR investigation of ASL has also demonstrated a high signal-to-noise ratio and high reproducibility utilizing pseudo-continuous ASL (pCASL) [12]. This is particularly important for the conduct of multisite clinical trials using fMRI findings as biomarkers of treatment responses, as the call for greater power [10] has led to the increasing utilization of multisite designs. Sutton et al. [11] showed that subject-to-subject variability was greater than 10 times that of site-to-site variability when using identical hardware and software in a 4-subject BOLD-based fMRI study.

Investigators in our group have shown that canonical resting state networks can be estimated from ASL data with similar components to BOLD based studies [13]. Furthermore, simultaneous BOLD and ASL fcMRI studies have shown that their respective functional connectivity (FC) values are correlated, supporting the use of ASL for these FC investigations [14]. However, Jann et al. [15] showed that although ASL FC values have acceptable repeatability, the overall ICC are low compared to BOLD in a study that utilized two different scanners, but these were located in the same facility and only separated in time by one day. These are significant weaknesses, as a practical multisite study would likely occur in at least two facilities spread out across the country with weeks to months between scans if the same subject was studies at different sites.

For their analysis, Jann et al. used Independent Component Analysis (ICA), a data-driven approach that allows the determination of the set of RSNs without the specification of specific seed regions, and therefore established the expected reliability coefficients for an ICA approach. However, studies of FC often test specific hypotheses about the relationships between RSNs using a seed-based analysis instead of ICA [16]. In seed-based analysis, the MRI signal from a specific brain region or set of voxels is used as the primary regressor, and the correlations to other areas of interest are determined.

In the present study, we investigated seed-based TRR in BOLD and ASL paradigms in 10 subjects to compliment and extend the Jann et al. findings across a longer time period in separate institutional facilities managed by two different groups. Each subject was scanned on two identical scanners running identical software. Both BOLD and pCASL images were collected during a block-design finger-tapping task and a resting state scan with analysis focusing on the areas related to the Default Mode Network (DMN) [17]. The ICC and Dice Similarity Coefficients (DSC) were determined and compared.

## Methods

### Subjects

#### Ethics approval and consent to participate

All study methods were carried out in accordance with the relevant guidelines and regulations according to the Declaration of Helsinki.

Subjects were ten healthy adults (5 males and 5 females) with an age range of 23 – 48 years old. All subjects were recruited in accordance with the Institutional Review Boards at the University of Pittsburgh and Massachusetts General Hospital. Written informed consent was obtained from all subjects prior to scanning at each location. For each subject the scans at both sites were conducted at approximately the same time of day. Subjects were advised to maintain the same caffeine intake on scan days and same sleep schedule the nights before. They were advised not to exercise on the day prior to scanning and on the day of scanning before the scan.

#### MRI scans

All images were collected on 3 T MAGNETOM Skyra MRI scanners (Siemens, Erlagen, Germany) with 70 cm Open Bore. Scanners were located at UPMC Children’s Hospital of Pittsburgh (PIT) and the Athinoula A. Martinos Center for Biomedical Imaging at Massachusetts General Hospital/Harvard-MIT Division of Health Sciences & Technology (MGH). Five of the subjects were scanned at PIT first, and five were scanned at MGH first. The mean time between scans was 109 days, the median was 83, and the range was 2 to 218.

A 32-channel head coil equipped with a rear-facing mirror was used for imaging at both sites. Task instructions were projected onto a screen at the rear of the bore. Task instructions were displayed using E-Prime 2.0 (Psychology Software Tools, Inc., Sharpsburg, PA). A blank screen was projected during resting-state and structural scans.

Scans were collected in the following order for each subject: (1) resting-state BOLD, (2) resting-state pCASL, (3) finger-tapping pCASL, (4) MPRAGE structural scan, (5) finger-tapping BOLD, (6) resting-state pCASL (repeat of #2), (7) finger-tapping pCASL (repeat of #3). For resting-state and structural scans, subjects were instructed to relax and keep their eyes open. For the finger-tapping task, subjects were instructed to hold their right hand against their chest and tap the thumb against the other 4 fingers in a 2–3-4–5-4–3-2 sequence at a rate of approximately 2 Hz. Tapping occurred in 20-s blocks cued by E-Prime display with pseudo-random ISIs of 15, 20, 25, or 30 s. A total of 10 tapping blocks occurred during each scan.

Two dimensional BOLD images were collected using an EPI sequence (field of view (FOV) of 196 mm × 196 mm, voxel size of 2.0 × 2.0 mm2 with 32 slices of 4.0 mm thickness in interleaved fashion with no slice gap) using a repetition time (TR) of 2.5 s, an echo time (TE) of 33 ms, and a flip angle of 80°. A total of 120 volumes were collected in each resting-state BOLD scan. Finger-tapping BOLD scans were 175 volumes at MGH and 190 volumes at PIT, although only the first 175 volumes were analyzed at each location. High-resolution structural images were collected for each participant using a T1-weighted scanning technique (3D MPRAGE sequence, TR/TE/Flip = 1.35 s/2.54 ms/9°; field of view = 256 mm × 256 mm; voxels size = 1.0 × 1.0 × 1.0 mm3; 144 slices per slab).

pCASL images were collected using an FOV of 256 × 256 mm2 and matrix of 64 × 64, yielding 4.0 × 4.0 mm2 voxels. 25 slices of 5 mm thickness were collected with no gap in an interleaved fashion with a TR of 3.8 s, a TE of 15 s, and a flip angle of 90°. The labeling duration was 1.48 s and the post-labeling delay was 1.2 s. A total of 92 volumes were collected in each resting-state pCASL scan, and 114 volumes were collected in each finger-tapping pCASL scan. This specific pCASL sequence has been used in previous fMRI studies [18–20].

### Image processing

All DICOM images were anonymized using custom Matlab (The MathWorks, Inc., v. 2018a) scripts and converted to NIFTI format for processing and analysis. Structural image origins were set to the anterior commissure using SPM 12 (http://www.fil.ion.ucl.ac.uk/spm/). Brain extraction was performed in SPM by segmenting the structural image for each subject and creating a brain mask by adding the segmented grey matter, white matter, and cerebrospinal fluid (CSF) images together with a threshold of 0.01. The brain mask was then applied to the functional BOLD and pCASL images to extract the brain.

### Finger-tapping fMRI analysis

BOLD and pCASL signal activation was calculated using FEAT analysis in FSL Version 5.0 (https://fsl.fmrib.ox.ac.uk/fsl/fslwiki/). 4D Brain-extracted images were preprocessed in FSL with MCFLIRT motion correction and spatial smoothing of 5 mm FWHM for BOLD images and 8 mm for pCASL images. Images were registered to the standard space MNI52 T1 2 mm brain atlas (built into FSL) using full 12 degrees of freedom affine transformation (translation, rotation, zoom, and sheer).

BOLD signal activation was modeled using the tapping task as the explanatory variable (EV), convolved with the default hemodynamic response function in FSL, a canonical double gamma function. Z-statistic BOLD images were rendered using a Z-threshold of > 2.3 and a corrected cluster significance threshold of p = 0.05. Site specific group maps of the BOLD images were calculated using FSL FEAT higher-level analysis with FLAME 1 mixed effects.

pCASL signal activation was modeled with 3 EVs: (1) control – tag baseline, (2) pseudoBOLD activation using the tapping task, and (3) perfusion activation. Positive and negative contrasts and F-tests were calculated for each EV. Z-statistic pCASL images were rendered with an uncorrected threshold of p = 0.05 on account of the reduced temporal resolution. Individual subject mean pCASL images were calculated for each scanning location using FSL FEAT higher-level analysis. Site-specific mean pCASL images were calculated using FEAT higher-level analysis with fixed effects.

Motor cortex regions of interest (ROIs) for the specific areas activated by our finger tapping task were generated so that comparisons of BOLD and pCASL signal change could be performed. This was done using all of the finger-tapping results (both BOLD and pCASL, as described above) as first level analyses for a group mean calculation in FSL FEAT. A Z-threshold of 20 was used to limit our comparison to only motor areas strongly activated in all scans. The resulting mean cluster image was converted to a mask image using SPM12’s Image Calculator function with a threshold of 0. This activated motor cortex mask was applied to all BOLD and pCASL Z-statistic images at the individual subject level. Signal change was then computed for each individual relative to their whole brain mean signal. Pearson correlation analysis of these signal change values was performed in SPSS (IBM SPSS Statistics for Windows, Version 22.0. Armonk, NY: IBM Corp.) on BOLD and pCASL signal change values using 95% confidence intervals.

### Resting state functional connectivity analysis

The CONN Functional Connectivity Toolbox (version 17.f, https://www.nitrc.org/projects/conn/) in Matlab was used for all functional connectivity analyses [21]. 4D BOLD images were put through the CONN default preprocessing pipeline where they were motion corrected, slice timing corrected, outlier scrubbed, segmented into white matter, gray matter, and CSF maps, normalized, and smoothed with an 8.0 mm Gaussian kernel. BOLD images were then denoised for white matter, CSF, and effect of rest. 4D label – control subtracted pCASL cerebral blood flow (CBF) images were created using the ASL toolbox [22] and preprocessing scripts provided by Chris Rorden (https://crnl.readthedocs.io/asl/index.html). CBF images were smoothed to 8.0 mm FWHM in the CONN toolbox and denoised for white matter and effect of rest.

ROI-to-ROI and Seed-to-Voxel weighted functional connectivity analyses were calculated for the preprocessed BOLD and CBF images using bivariate correlation and hemodynamic response function weighting. Using the atlas provided within Conn, the DMN subregions medial prefrontal cortex (MPFC), posterior cingulate cortex (PCC), and left and right lateral parietal lobe (LLP and RLP, respectively) as well as the Anterior Cingulate Cortex (ACC) and right and left Anterior Insula (based on their involvement in pain scans and our interest in using these areas for future analysis) were used as seed regions. All functional connectivity images were thresholded at 0.25.

Intraclass correlation coefficients (ICC) [23] were calculated for the functional connectivity Z-scores for each seed region using custom Matlab scripts and the Matlab IPN toolbox developed by Xi-Nian Zuo [24, 25]. Negative ICC values are known to be difficult to interpret and were changed to zero [26]. Multivariate repeated measures ANOVA was used to compare functional connectivity Z-scores for the MPFC and PCC seed regions in the DMN at all ROIs using scanning location as the within-subjects variable. Additionally, Dice Similarity Coefficients (DSC) [15, 27, 28] were calculated for 3D functional connectivity image matrices. The DSC quantifies the spatial overlap for two or more images, ranging from 0 (no spatial overlap) to 1 (indicating complete overlap). DSC’s were generated by the CONN toolbox comparing BOLD and pCASL resting state images collected at MGH with images collected at PIT.

To further analyze the DMN in the seed-based pCASL resting state scans, a second analysis was done which used ICA to determine the data-derived DMN locations, and the above analysis was then repeated. This is referred to as dual-regression fcMRI [25]. To accomplish this, two separate ICA runs were performed; one for each pCASL set at each of the two sites. All 10 subjects’ data for the site were entered into CONN Toolbox. Again, white matter signal and the effect of rest were removed during denoising. To ensure that artifactual independent components were not identified (the DMN is robustly connected at rest and is typically easy to identify), 50 components were used. The component that appeared to best represent the DMN thorgh manual visual inspection was selected and thresholded at Z = 2. Each area of the DMN was then isolated from that component in individual masks and fed back into CONN for a ROI-to-ROI analysis using the ICA-defined PCC as the seed. The rest of the analysis mirrored that described for pCASL above.

### Cerebral blood flow analysis

CBF maps were generated for each pCASL image set using the ASL toolbox and Rorden preprocessing scripts described above. Mean CBF maps were generated for each subject at each scanning location using SPM12. CBF maps were generated for the resting state scans and separately for the tapping and resting portions of the finger-tapping scans. CBF maps were generated in SPM12.

Global mean CBF was calculated from these mean CBF maps using custom Matlab scripts. CBF values for the motor cortex ROI were calculated by applying the motor cortex mask described above to each mean CBF image using FSLSTATS. Repeated measures ANOVA analyses were performed in SPSS to compare CBF values between the two scanning locations.

All data collected for this study, including physiologic signals where available, has been uploaded for public sharing on OpenNeuro (https://openneuro.org).

## Results

### Finger-tapping task

**Fig. 1:**
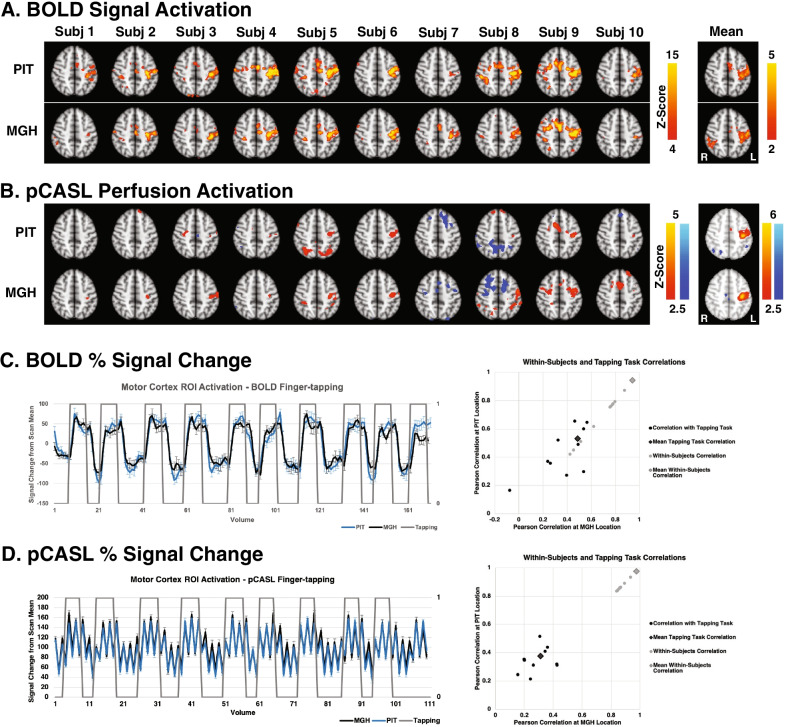
Activation measured in BOLD and pCASL finger-tapping scans at PIT and MGH scanning locations. Rendered Z-statistic images for each subject at each location for BOLD (**a**) and pCASL perfusion activation (**b**). Negative perfusion change is shown in blue, and positive is shown in red/yellow. Both BOLD and pCASL scan sequences showed activation in the motor cortex, and group mean images at each location were very similar. Signal changes for BOLD scans (**c**) at each location (PIT = blue, MGH = black) were significantly correlated between the two locations and with the finger-tapping task, shown in gray. Individual subject Pearson correlations with the task and within-subject are shown to the right, demonstrating excellent repeatability. Signal changes for pCASL scans (**d**) were significantly correlated with the tapping task at PIT and at MGH, and were significantly correlated between the two scanning locations, again as shown to the right

Both BOLD (Fig. 1a) and pCASL scans (Fig. 1b) showed strong activation in the motor cortex at both scanner locations. In the BOLD scans, signal change in the motor cortex showed significant Pearson correlations with the finger-tapping task at both the PIT (0.539, p < 0.001) and MGH (0.490, p < 0.001) scan locations (Fig. 1c), and showed a strong correlation of 0.944, p < 0.001 between the two locations. In the pCASL scans, percent signal change in the motor cortex showed a significant Pearson correlation with the tapping task at PIT (0.375, p < 0.001) and at MGH (0.312, p < 0.001) (Fig. 1d). Additionally, percent signal change was significantly correlated between the two locations for the pCASL scans (0.976, p < 0.001).

### Resting state functional connectivity

**Fig. 2:**
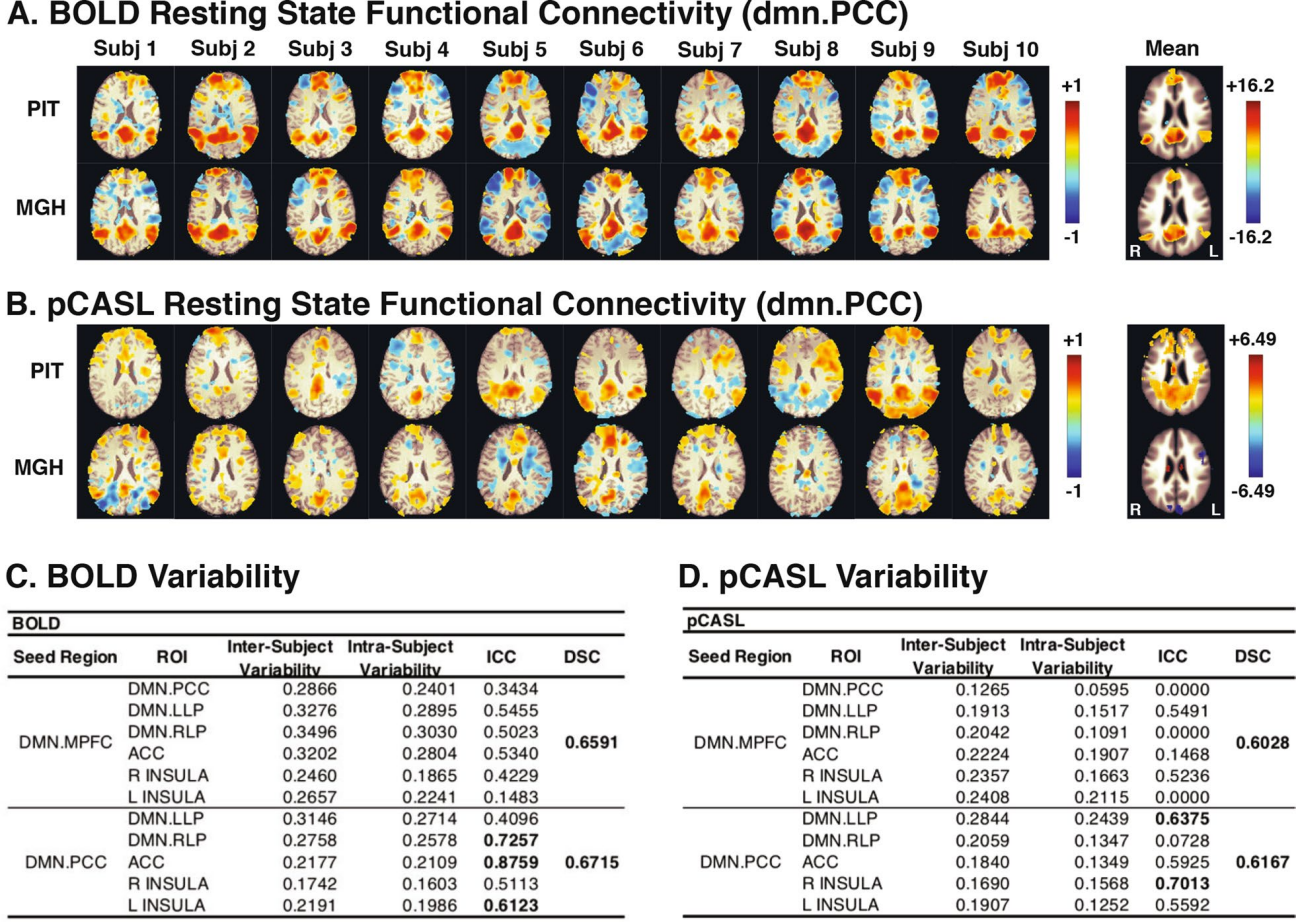
Default mode network functional connectivity in BOLD and pCASL scans at both scanning locations. Functional connectivity maps with PCC as the seed region are shown for each subject at each location for BOLD (**a**) and pCASL (**b**) resting state scans. Inter- and intra-subject variability expressed as mean standard deviations, intraclass correlation coefficients (ICC) and Dice Similarity Coefficients (DSC) for BOLD (**c**) and pCASL (**d**) resting state scans are shown for the DMN subregions MPFC, PCC, LLP, and RLP as the seed regions and the PCC, LLP, RLP, ACC, and left and right insula as the ROIs. Inter- and intra-subject variability were not significantly different for both BOLD and pCASL scans. Both the BOLD and pCASL functional connectivity maps showed high intraclass correlation in at least one DMN pathway, but the pathways showing the highest ICC values differed between the two scan sequences

Functional connectivity analyses with the PCC as the seed region identified elements of the DMN in both the BOLD (Fig. 2a) and pCASL scans (Fig. 2b) at both locations, although activation was clearer and more consistent between subjects and locations in the BOLD images, while the DMN is not clearly identified in the PCC seed-based pCASL images. Nonetheless, high intraclass correlations between the PIT and MGH locations were found for DMN connectivity in both types of scans. In the BOLD scans (Fig. 2c), high intraclass correlations were found for PCC to RLP (0.726), ACC (0.876), and left insula (0.612), for the LLP to RLP (0.617), ACC (0.608), and left insula (0.689), and for the RLP to ACC pathways (0.606). In the pCASL scans (Fig. 2d), high intraclass correlations were found for the PCC to LLP (0.638) and right insula (0.701).

Multivariate repeated measures ANOVA showed that functional connectivity Z-scores for both BOLD scans did not have significant variability for location, but did have significant variability between subjects (Fig. 2c). In other words, variability was greater between subjects than between locations. For BOLD resting state functional connectivity Z-scores, within-subjects variability was not significantly different for both the MPFC seed region, F(4, 6) = 2.431, p = 0.205, and the PCC seed region, F(4, 6) = 5.546, p = 0.06. For BOLD scans, between-subjects variability was significantly different for both the MPFC seed region, F(4, 6) = 62.333, p = 0.001, and for the PCC seed region, F(4, 6) = 54.616, p = 0.001.

Similar to the BOLD scans, the pCASL resting state scans did not have significant within-subjects variability for either the MPFC seed region, F(2, 6) = 3.611, p = 0.233, or the PCC seed region, F(2,6) = 1.545, p = 0.443 (Fig. 2d). Between-subjects variability for pCASL scans was not significant for the MPFC seed region, F(2, 6) = 2.786, p = 0.288, but was significant for the PCC seed region, F(2, 6) = 97.824, p = 0.01.

Dice Similarity Coefficients were higher for BOLD resting state scans (Fig. 2c) than for pCASL resting state scans (Fig. 2d). For the MPFC, PCC, LLP and RLP seed region, the DSC was 0.659, 0.672, 0.667, and 0.664 respectively for the BOLD scans, while the pCASL scans had DSC values of 0.603, 0.617, 0.473 and 0.451 in these regions. Generally, ICC and DSC coefficients greater than or equal to 0.6 are considered to be at least “good” correlations [29].

**Fig. 3:**
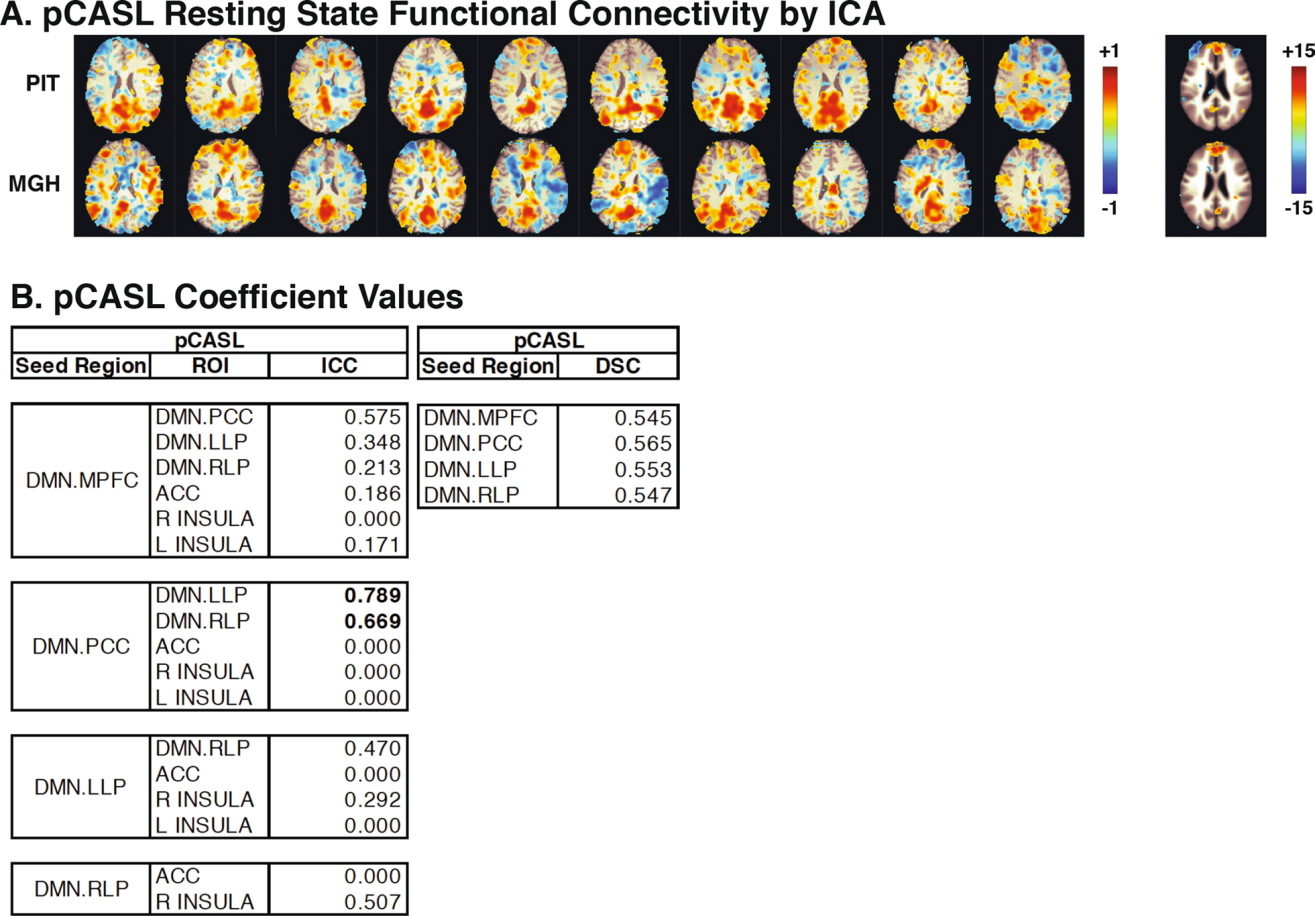
Dual regression default mode network functional connectivity for pCASL scans at both scanning locations. Functional connectivity maps with the ICA defined PCC as the seed region are shown for each subject at each location for pCASL (**a**) resting state scans. Inter- and intra-subject variability expressed as mean standard deviations, intraclass correlation coefficients (ICC) and Dice Similarity Coefficients (DSC) for pCASL (**b**) resting state scans are shown for the DMN subregions MPFC, PCC, LLP, and RLP as the seed regions and the PCC, LLP, RLP, ACC, and left and right insula as the ROIs. Overall the intraclass correlations increased as a result of the ICA ROI selection, with specific improvements in the DMN pathways

The results of the ICA-based analysis are displayed in Fig. 3a. The DMN in each subject’s map is much more clearly defined when compared to the seed-based pCASL resting state connectivity maps in Fig. 2. The group map for each location shows a much cleaner picture of DMN connectivity, and the two locations resemble each other more closely. As with the seed-based pCASL analysis, the pCASL resting state scans did not have significant within-subjects variability for either the MPFC seed region, F(4, 6) = 1.944, p = 0.271, or the PCC seed region, F(4,6) = 3.878, p = 0.105. Between-subjects variability for pCASL scans was significant both for the MPFC seed region, F(4, 6) = 32.302, p = 0.002 and for the PCC seed region, F(4, 6) = 34.730, p = 0.002.

Although ICA-based analysis produced cleaner images of DMN functional connectivity, the correlation coefficients were similar or slightly worse compared to ICC and DSC values for seed-based analysis (Fig. 3b). ICC values for ICA-based pCASL images were only above the threshold of 0.6 for two pathways: PCC – LLP, with an ICC = 0.789, and PCC – RLP, ICC = 0.669. No DSC values were above the 0.6 threshold for ICA-based pCASL images.

### Cerebral blood flow

**Fig. 4:**
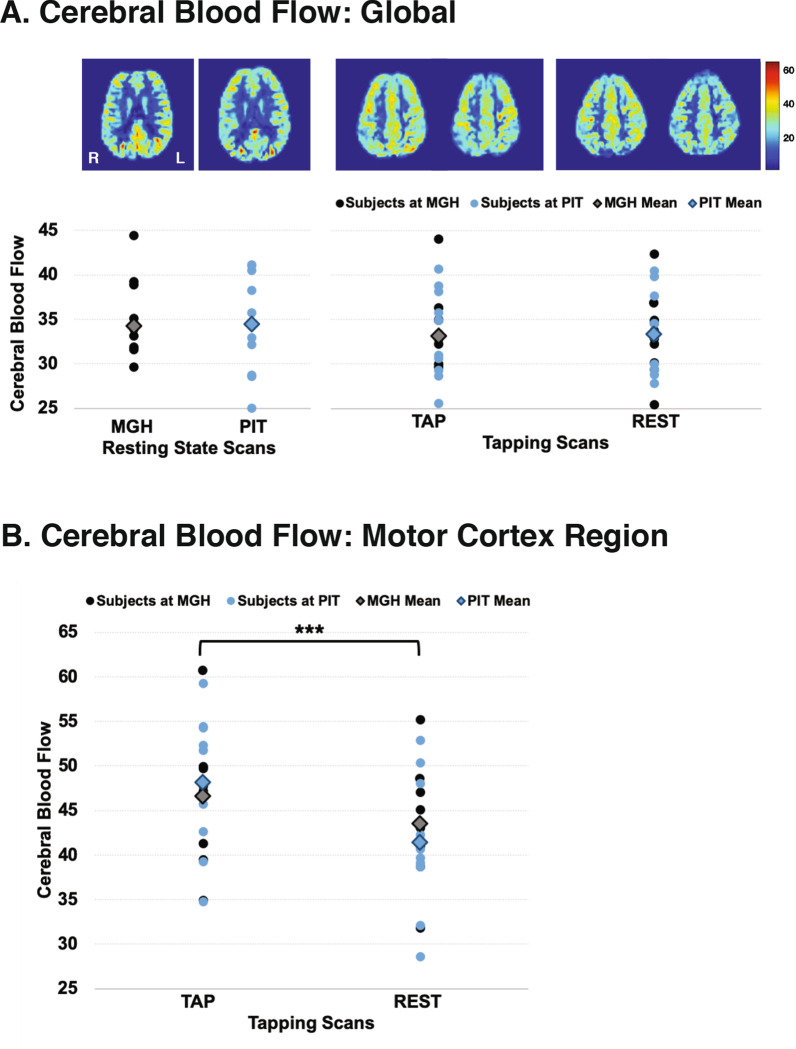
Global and regional cerebral blood flow (CBF) measured at the two scanning locations. Mean CBF maps for each subject at each location for resting state scans (**a**) and for finger-tapping scans (**b**). CBF maps for the finger-tapping scans are shown separately for the tapping and resting portions of the scans. Mean global CBF and regional flow values in the motor cortex compared with paired-samples t-tests (**c**) were not significantly different between the two scanning locations for the resting state scans or for the tapping and resting portions of the finger-tapping scans

Global CBF was very similar between the two scanning locations (Fig. 4a), and repeated measures ANOVAs showed no significant differences between the two scanning locations. Mean (SD) global CBF during resting state scans was 34.26 (5.56) at MGH and 34.45 (5.83) at PIT, F(1, 9) = 0.009, p = 0.925. Mean global CBF during the resting portion of the finger-tapping scans was 33.27 (4.55) at MGH and 32.78 (5.32) at PIT, F(1, 9) = 0.087, p = 0.775. Mean global CBF during the tapping portion of the finger-tapping scans was 33.12 (5.47) at MGH and 33.37 (5.00) at PIT, F(1, 9) = 0.027, p = 0.874.

Regional flow in the motor cortex was higher during the finger-tapping scans compared to rest once the hemodynamic delay was taken into account (Fig. 4b). Differences were not found between the MGH and PIT scanning sites (F(1,9) = 0.21, p = 0.89), but motor cortex CBF did, as expected, vary between tapping and resting tasks (F(1,9) = 41.77, p < 0.001). Mean motor cortex flow during the resting portion of the finger-tapping scans was 43.56 (6.22) at MGH and 41.42 (7.57) at PIT, F(1, 9) = 2.704, p = 0.134. Mean flow during the tapping portion of the scans was 46.64 (7.00) at MGH and 48.16 (7.64) at PIT, F(1, 9) = 3.112, p = 0.112.

## Discussion

A noticeable gap in the literature has been the lack of comparison between BOLD and pCASL functional brain imaging modalities related to activation and connectivity patterns. In particular, comparisons between scanners in different locations and scanning sessions separated by longer periods of time are missing. The present study addresses these issues by demonstrating in healthy normal subjects that while BOLD shows greater test–retest repeatability, pCASL is acceptable as well, including during the performance of a finger tapping task. pCASL also demonstrated consistent measures of global CBF across scanners and between days. Overall, the variability between subjects was much greater than the variability between scanners and days. These findings are salient to the design and execution of longitudinal translational studies and clinical trials in which brain fMRI findings are increasingly being tested as potential biomarkers of disease and/or treatment responses.

Prior studies have explored the test–retest repeatability of BOLD and of pCASL images. In a multi-scanner BOLD imaging study with four subjects, Sutton et al. found that subject-to-subject variance was nearly 10 times greater than the site-to-site variability [11]. Chen et al. examined twelve subjects using ASL and expanded the time frame out to one week, finding that pCASL’s reliability was superior to both pulsed and continuous ASL [12]. As mentioned, Jann et al. investigated both ASL and BOLD in a study of 10 subjects on two scanners in the same facility, with scans separated by one day. They found that between scanner ICC’s averaged 0.89 for BOLD and 0.58 for pCASL [15]. The results of the above show that both BOLD and pCASL could be used for reliable mapping of the brain’s resting state networks across narrow periods of time and space, but evidence that this could be done across months in completely separate facilities was lacking.

Our study adds to the literature by providing practical values for TRR data in both BOLD and pCASL functional brain imaging utilizing task-based and resting state paradigms across two unique conditions. First, we utilized scanners in completely different research centers in different cities, which were maintained with separate quality assurance procedures by local staff. This approach mirrors the most frequent situation in multi-site treatment studies for many medical conditions. Second, we used realistic time frames for a multisite treatment study for re-testing, with a mean of 109 days between scans (median 83). Both of these issues can explain the decreased ICCs we found as compared to Jann et al., as could their use of concatenated data sets across both BOLD and pCASL for their ICA analysis. More importantly, because the ICC and DSC values in our study were still considered acceptable despite these additional variables, our results suggest that both BOLD and pCASL can be used for reliable and repeatable imaging across multiple sites and long interscan periods of time. For both image types throughout this study, between-subject variability is consistently higher than the between-site variability, providing further evidence that multisite studies with both BOLD and pCASL techniques are feasible and scientifically justified.

The comparison of BOLD to pCASL in the present study also offers unique insight into the strengths of each scanning method. For the investigation of task-related brain activity in BOLD and pCASL imaging, it is obvious from Fig. 1 that activation in any individual subject is more robust for BOLD imaging than for pCASL. This is not surprising given the overall susceptibility to noise (from physiologic, motion, and image subtraction sources) found in the pCASL images and the fact that there are at least twice as many BOLD scans collected in each scanning period due to the longer TR and image subtractions necessary for pCASL analysis. Motor cortex activity is visible in each BOLD scan, while the majority of pCASL individual scans do not show activation that reaches threshold in that area. As expected, the pCASL group map does show clear motor cortex activity, and it is notable that it appears more localized than the BOLD maps which show activity across the motor and sensory strips of the central gyrus. It is our opinion that if individual scans or subjects collected over short time frame are the outcome of interest, then BOLD may be the preferred method. However, if scanning times can be lengthened to account for the longer effective TR and group results strongly correlated to a more specific area are the focus, then pCASL would perhaps be the technique of choice.

The seed-based resting state functional connectivity for both the BOLD and pCASL images in Fig. 2 showed acceptable DSC values, suggesting that the results were consistent across sites. Likewise, the ICC values, especially for the seed region of the PCC, also suggested that differences due to scanning site and elapsed time were minimal. Visually, both the BOLD and pCASL individual and group maps show consistency between the scanning sites, but the BOLD technique produces cleaner DMN activity, especially in the group maps.

The group representation of the DMN via correlation with the PCC for the pCASL technique in Fig. 2 was lower than expected. When ICA was used to determine the DMN in the pCASL images using a data-driven approach (instead of using CONN’s built-in atlas to identify the PCC), there was substantial improvement (Fig. 3). Visually, the DMN became apparent in each subject’s scans as well as in the group average’s images. The Z-scores for the group maps greatly increased as well, from a maximum of 6.49 to 15, as can been seen by comparing the legends for the two figures. From the ICC values, there was an increase in specificity for the DMN as well. The fact that DSC showed little, if any, changes suggests that is not sufficient as a singular measure for investigations of repeatability. When using ICA, the majority of ICC values between the PCC and the mPFC improved, as expected. We would caution against over-interpretation of the individual ICC values between each region; instead we have chosen to consider the trends. Overall, the pCASL group results in Fig. 1 showed specific activation in the motor cortex; likewise Fig. 3 shows specificity to the DMN when using ICA for the resting state images. The data-driven ICA dual regression technique is common in pCASL analysis, and our results suggests that it improves repeatability and should continue to be used.

During both resting and tapping scans, global CBF is consistent and showed excellent reliability with other studies [30, 31]. From both the CBF images and from the repeated measures ANOVA results, subject-to-subject variation was greater than site-to-site, and we found no consistent site-to-site difference. This is not surprising, since identical scanner hardware and reconstruction/analysis software were used, but this has also been shown in studies that used different scanner platforms across multiple sites [32]. Likewise, CBF means and repeated measures ANOVA testing suggest that regional CBF is significantly increased in the motor cortex during tapping and decreased during rest phases, and those differences can be replicated in the same subjects at different scanning locations.

The use of the same pulse sequences on the same scanner model at both sites undoubtedly aided our findings, as we were able to ensure that all elements were as close as possible between the sites. This might not be the case if different scanners, sequences, and/or settings were being used at each site. Regarding study limitations, similar to Friedman et al. [7], we found that performing a multisite study requires intense effort and a focus on many factors that can affect the site-to-site results. Despite efforts to make things identical between sites, several differences were found that affected the analysis of the data. For example, one scanner saved the first 3 images of each scan, and the second scanner automatically discarded these scans. Although this seems like a trivial issue, it did affect the timing of the tapping task related to the image sequence across the two locations and had to be accounted for. Another issue encountered was that one scanner initially used dynamic fields of view, whereas the other scanner used fixed FOVs. Discovery of this issue necessitated re-scanning some subjects at one location so that the FOVs matched at both sites. Small, but significant, inconsistencies in ancillary equipment, such as physiologic monitoring devices, also limited our study, highlighting the necessity to ensure that aspects are thoroughly protocolized. It should further be noted that had they been available for all scans, use of the physiologic monitor data would be expected to be of greatest benefit to the pCASL images, suggesting that the results we present may be considered a “worst-case scenario” for the pCASL images and further strengthen the opinion that pCASL shows acceptable repeatability across sites and scanners. Finally, we should mention that test–retest studies such as this should be repeated as technology advances and improvements are made to scanner hardware and data acquisition systems. For example, the wide-adoption of parallel or simultaneous multislice imaging techniques can alter either temporal or spatial resolution, which could have large effects on the measures used in our paper.

## Conclusion

Multisite and longitudinal studies of brain activity/connectivity during a task or at rest are possible and provide reliable results using both BOLD and pCASL sequences. As expected, BOLD signal appears more robust; however, the pCASL signal may be more specific to a particular brain region, shows improvement when an ICA approach is used, and is able to provide reliable quantitative measurements of blood flow. Care should be taken to ensure that small differences in scanner settings are taken into account by using scientists from both (or all) institutions for study design and data collection and analysis.

## Data Availability

The datasets used and/or analysed during the current study are available from the corresponding author on reasonable request.

## Abbreviations

TRR: Test–retest reliability and reproducibility
fMRI: Functional magnetic resonance imaging
BOLD: Blood oxygen level dependent
FcMRI: Functional connectivity
rs-fcMRI: Resting State Fcmri
ICC: Intraclass correlation coefficients
Rsns: Resting state networks
ASL: Arterial spin labeling
Pcasl: Pseudo-continuous
FC: Functional connectivity
ICA: Independent component analysis
DMN: Default mode network
DSC: Dice similarity coefficients
PIT: UPMC Children’s hospital of pittsburgh
MGH: Athinoula A. Martinos Center for Biomedical Imaging at Massachusetts General Hospital/Harvard-MIT Division of Health Sciences & Technology
MPFC: Medial prefrontal cortex
PCC: Posterior cingulate cortex
LLP: Left lateral parietal lobe
RLP: Right lateral parietal lobe
ACC: Anterior cingulate cortex
